# Antibiotic and Antibiofilm Activities of *Salvadora persica* L. Essential Oils against *Streptococcus mutans*: A Detailed Comparative Study with Chlorhexidine Digluconate

**DOI:** 10.3390/pathogens9010066

**Published:** 2020-01-16

**Authors:** Merajuddin Khan, Hamad Z. Alkhathlan, Shams Tabrez Khan

**Affiliations:** 1 Department of Chemistry, College of Science, King Saud University, P.O. Box 2455, Riyadh 11451, Saudi Arabia; mkhan3@ksu.edu.sa (M.K.); khathlan@ksu.edu.sa (H.Z.A.); 2 Department of Agricultural Microbiology, Aligarh Muslim University, Aligarh 2002002 UP, India

**Keywords:** essential oils, *Salvadora persica* L., antibiotic resistance, *S. mutans*, oral pathogens, Chlorhexidine

## Abstract

The use of organic components from plants as an alternative antimicrobial agent is becoming popular due to the development of drug-resistance in various pathogens. Essential oils from fresh (MF-1) and dried (MD-1) roots of *Salvadora persica* L. were extracted and benzyl isothiocynate was determined as their chief constituent using GC-MS and GC-FID. The antibiofilm and antimicrobial activities of MD-1 and MF-1 against *Streptococcus mutans* a dental caries causing bacteria were determined using multiple assays. These activities were compared with chlorhexidine digluconate (CHX) and clove oil, well known antimicrobial agents for oral hygiene. Essential oils demonstrated IC_50_ values (10–11 µg/mL) comparable to that of CHX, showed a significant reduction (82 ± 7–87 ± 6%) of the biofilm formation at a very low concentration. These results were supported by RT-PCR studies showing change in the expression levels of *Atl*E, *gtf*B, *ymc*A and *sod*A genes involved in autolysis, biofilm formation and oxidative stress, respectively. The results presented in this study show the robust bactericidal and antibiofilm activity of MD-1 and MF-1 against *S. mutans* which is comparable to Chlorhexidine digluconate. Our results suggest that these essential oils can be as effective as CHX and hence can serve as a good alternative antimicrobial agent for oral hygiene.

## 1. Introduction

*Salvadora* is a small genus of evergreen trees or shrubs found mainly in hot and dry areas of Asia and tropical Africa. It belongs to the family *Salvadoraceae*, a small plant family comprising of about three genera and a total of 11 known species [1,2]. Among these, *Salvadora persica* L. is one of the most important plant species as it is famous for its applications in oral hygiene and various other ethnobotanical uses [3]. *S. persica* L., native to middle East and Africa, is a small well branched shrub with greenish yellow flowers and small red fruits when ripe. It grows up to a maximum height of 6–7 m with greenish small leaves (3.8–6.3 by 2–3.2 cm), having a robust aroma of cress or mustard [4,5]. *S. persica* L. roots/stems are known with various names like Mustard tree, Miswak, Koyojii and Pilu in Gulf region, Japan and India, respectively and have been traditionally used for maintaining oral hygiene for centuries [3,6]. In addition to extensive use in oral hygiene, other organs of the plants, such as the stems, leaves, flowers, roots, fruits and twigs have other wide spread traditional applications. For example, several organs of *S. persica* L. have been used for the remedy of various illnesses, including piles, urinary disorder, rheumatism, skin diseases, seasonal cough and cold, diabetes, asthma, gout, constipation and respiratory infections [3].

Recently, aqueous extracts of *S. persica* L. root have been effectively utilized for the synthesis of different nanoparticles (NPs) which have shown attractive microbicidal and catalytic activity for the Suzuki coupling reactions [7,8]. Moreover, the pharmacological properties of *S. persica* L. are widely known and several fascinating biological activities, including antimicrobial, antitumor, hypoglycemic, anti-osteoporosis, anti-inflammatory, analgesic, antiulcer, antioxidant, enzyme inhibitory, anticonvulsant and sedative activity have been reported for this plant [3].

Although various pharmacological properties of *S. persica* L., along with its applications in folk medicines for the remedy of several diseases, have been reported, *S. persica* L. is most widely used and known for maintaining oral hygiene. One reason may be the remarkable antimicrobial activities of various compounds from *S. persica* L. [9,10,11]. Many of these reports evaluated the microbicidal properties of *S. persica* L. against different microorganisms. Yet, a detailed report on the drying effects on the chemical constituents of *S. persica* L. essential oil (EO) and their effects on antimicrobial activity are lacking. Furthermore, oral pathogens are a matter of greater concern than previously thought as their role in other diseases is becoming clear [12]. Moreover, the development of drug resistance in bacteria requires minimizing the use of synthetic chemotherapeutic agents and promote the use of plant-based products as alternative antimicrobial agent [13,14]. One of the most widely used chemotherapeutic agents for oral hygiene is chlorhexidine digluconate (CHX). However, it is also known to be associated with a number of disadvantages, such as bitter taste, taste alteration for at least 4 h and tooth discoloration [15]. Moreover, clove oil extracted from flower buds of *Syzygium aromaticum*, a plant species of the family *Myrtaceae* has been popularly used for toothache and maintaining oral hygiene in traditional medicine. However, detailed comparative studies on the antimicrobial activities of clove oil, CHX and *S. persica* L. oils are lacking. This paper reports on the chemical determination of EO components of fresh and dried *S. persica* L. roots and undertakes a detailed comparative study on the antimicrobial activity of these EOs with those of commercially used antiseptic agents CHX and clove oil against a well-known oral pathogen *Streptococcus mutans*. Here, we report, for the first time, a comparative study of *S. persica* L. essential oils, clove oil and CHX against *S. mutans* and their possible mechanism of actions against the test organism.

## 2. Results

### 2.1. Chemical Composition of Essential Oils

**Figure 1:**
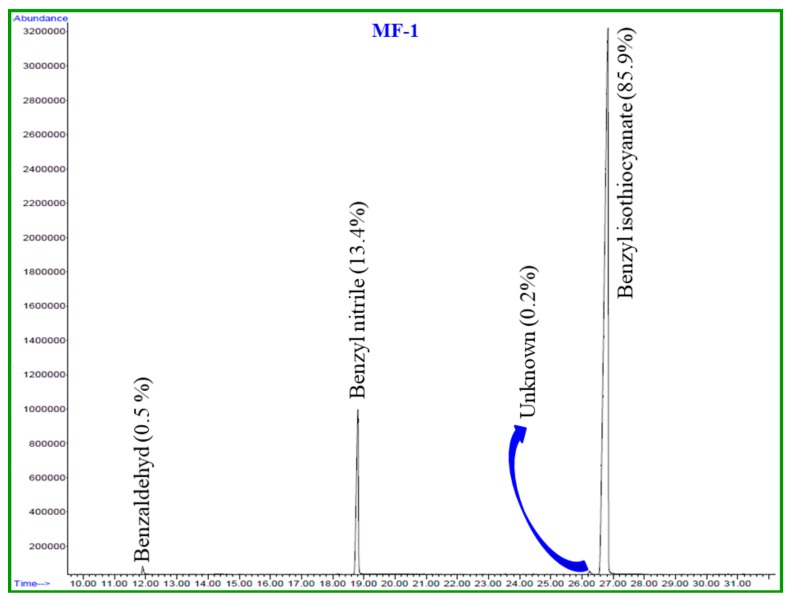
GC-FID chromatogram of EO constituents of the fresh *S. persica* L. roots on HP-5MS column.

**Figure 2:**
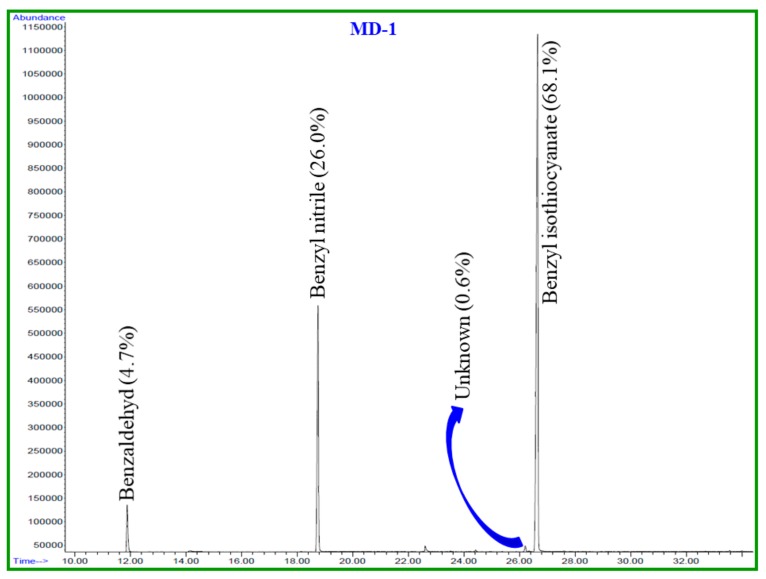
GC-FID chromatogram of EO constituents of the dried *S. persica* L. roots on HP-5MS column.

This paper identifies, in detail, the chemical constituents of EOs extracted from fresh and dried *S. persica* L. roots. The hydro-distillation of fresh and dried roots of *S. persica* L. produced light yellow oils in yields of 0.2% and 0.03%, *w*/*w*, respectively. The comparison of the oil yield of fresh and dried roots of *S. persica* L. in the present study revealed that fresh roots contained about a seven times higher oil yield than those of the dried roots. GC-MS and GC-FID housing a nonpolar column HP-5MS (Agilent Technologies, Santa Clara, CA, USA) were used to determine the phytochemical constituents of these oils. A total of four components were detected from both oils. Based on mass fragmentation pattern, retention time, and calculated linear retention index values, three components from each essential oil were identified, which account for 99.7% and 98.8% of the total oils of fresh and dried *S. persica* L. roots, respectively. The identified chemical constituents from fresh and dried roots of *S. persica* L. and their relative quantity (%) are detailed in Table 1 according to their order of elution through the HP-5MS column. Moreover, a GC–FID chromatogram of fresh and dried of *S. persica* L. roots EOs is given in Figure 1 and Figure 2, respectively.

**Figure 3:**
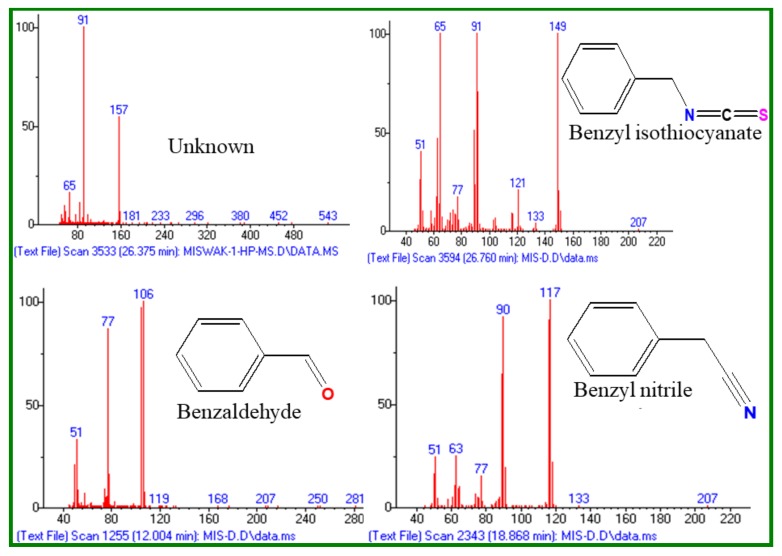
EIMS of essential oil constituents of the fresh and dried *S. persica* L. roots.

The main volatile components identified from the fresh and dried roots of *S. persica* L. were: benzyl isothiocyanate (LRI 1373, 85.8% and 68.1%); benzyl nitrile (LRI 1140, 13.4% and 26.0%) and benzaldehyde (LRI 958, 0.5% and 4.7%), respectively (Figure 3). From Table 1, it is clearly evident that the chemical components of the fresh and dried roots of *S. persica* L. were almost identical. However, the content of each constituent differed significantly in both oils. For instance, the content of benzyl isothiocynate, the most abundant compound of *S. persica* L. oils, was found to be about 18% less in the essential oil of dried *S. persica* L. roots. On the contrary, it was noticed that the contents of other constituents, such as benzaldehyde and benzyl nitrile were 4%–13% higher in the essential oils of dried *S. persica* L. roots. Thus, this study reveals that drying of *S. persica* L. roots at room temperature has prominent effects on the quantitative composition of *S. persica* L. roots essential oils.

### 2.2. Antimicrobial Activity of Compounds

#### 2.2.1. Microdilution Method

In the microdilution method, the maximum microbicidal activity against *S. mutans* was exhibited by CHX, where 6 μg/mL of the compound completely inhibited the growth (Table S1). The EOs (MD-1 and MF-1) prepared from *S. persica* L. exhibited remarkable antimicrobial activity against *S. mutans*. In fact, these activities were comparable to CHX widely used in oral hygiene products as an antiseptic agent. The growth of *S. mutans* was completely inhibited by 8 μg/mL of MF-1, while a complete growth inhibition of *S. mutans* by MD-1 was detected at a concentration of 10 μg/mL (Table S1). No significant antimicrobial activity of clove oil was detected as 14 μg/mL of clove oil the highest test concentration inhibited the growth of *S. mutans* by only 16%.

#### 2.2.2. Spread Plate Method

**Figure 4:**
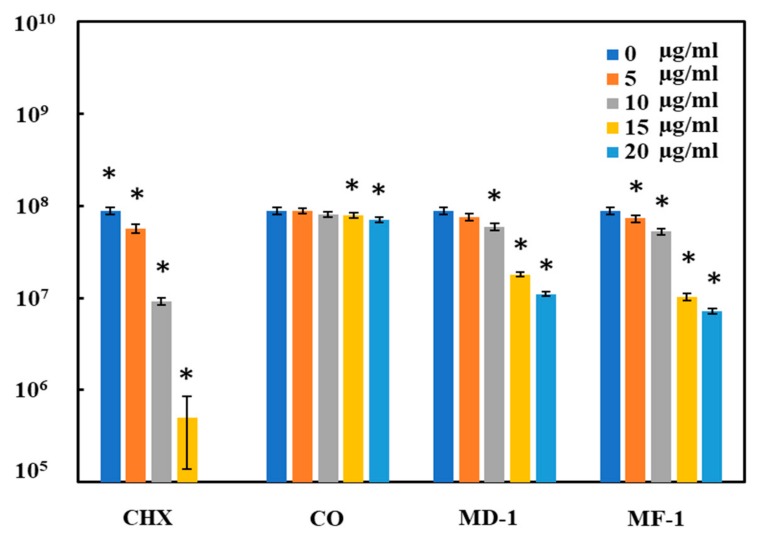
*S. mutans* growth inhibition with various concentrations (0, 5, 10, 15 and 20 µg/mL) of chlorhexidine digluconate (CHX), clove oil (CO), and essential oils from *S. persica* L. (MD-1 and MF-1). The experiments were carried out in triplicate and the values are shown as mean ± S.D error. Significant values (*p*-values > 0.05) are marked with an asterisk (*).

The test compounds exhibited almost a similar pattern of antimicrobial activity when tested using the spread plate method. CHX exhibited the maximum microbicidal activity against *S. mutans* with an IC_50_ value of only 6 μg/mL. CFU counts decreased by 90% at a concentration of 10 μg/mL of CHX, while MD-1 and MF-1 also exhibited comparable activities, showing IC_50_ values of 11 and 10 μg/mL, respectively (Figure 4). A 92 ± 7% and 87 ± 6% decrease in CFU counts was observed when *S. mutans* was grown with 20 μg/mL of MF-1 and MD-1, respectively. An almost negligible antimicrobial activity of clove oil was observed, as only a 19 ± 5% decrease in CFU counts was seen, even with the maximum test concentration of 20 μg/mL.

### 2.3. Change in Bacterial Viability

#### 2.3.1. Viability Assay Using MTT

**Figure 5:**
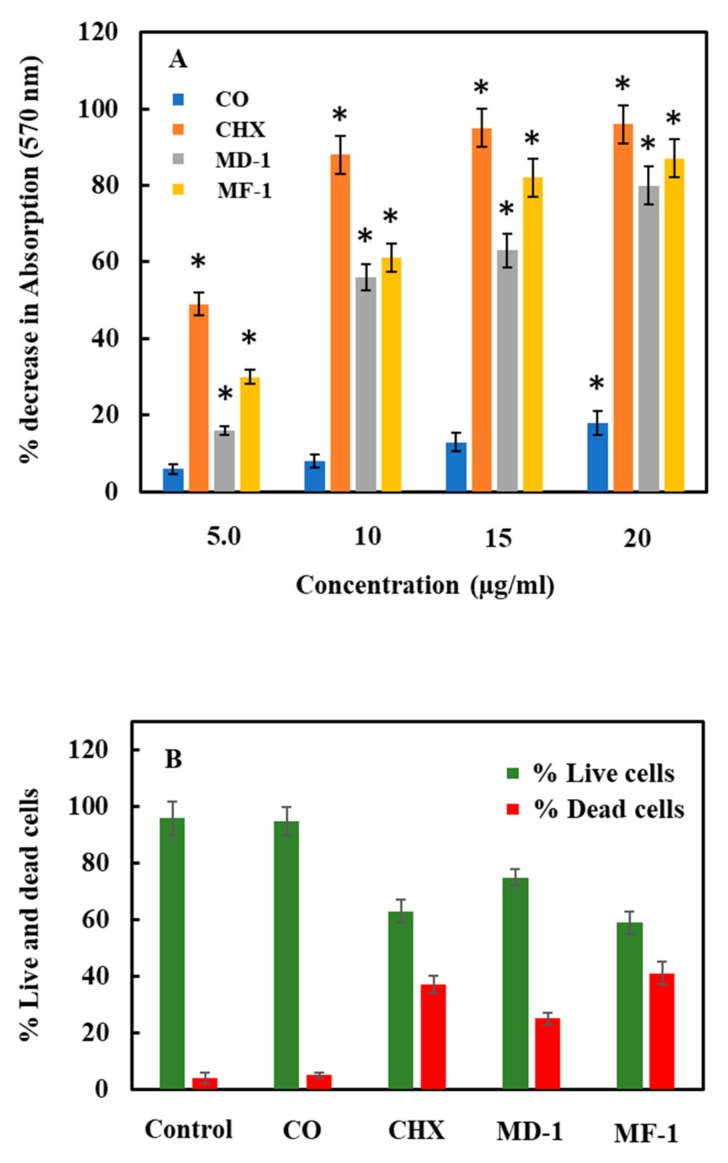
Decrease in the viability of *S. mutans* when grown with various concentrations of Chlorhexidine digluconate (CHX), clove oil (CO) and essential oils (MD-1 and MF-1) as determined by MTT assay and through (**A**). Significant values (*p*-values > 0.05) are marked with an asterisk. Panel (**B**) shows the % population of Live and Dead as determined by BacLight staining.

*S. mutans* was grown with 5, 10, 15 and 20 μg/mL of the test compounds. The change in the viability as found in MTT assay is shown in Figure 5a. MF-1 and MD-1 decreased the cellular viability by 80 ± 5% and 82 ± 7, respectively, with a maximum test concentration of 20 μg/mL. CHX caused the highest decrease in the metabolic activity with a 95 ± 5% decrease in the absorption value at a concentration of 15 μg/mL, whereas only a decrease of 18 ± 6% in the viability was obtained with the highest test concentration of clove oil.

#### 2.3.2. Live and Dead Staining

**Figure 6:**
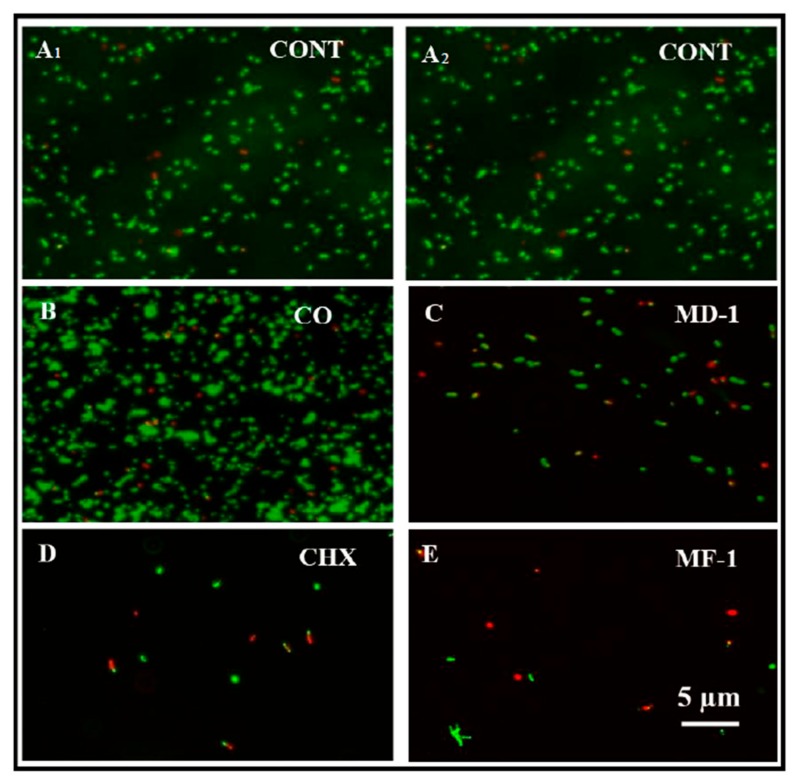
Decrease in the viability of *S. mutans* when grown with 5 µg/mL of Chlorhexidine digluconate (D; CHX), clove oil (CO; B) and essential oils (MD-1; C and MF-1; E) as determined through Live and Dead staining. Panel A1 and A2 show untreated control.

*S. mutans* was grown in the presence of 5 µg/mL of MD-1, MF-1, clove oil and CHX for 8 h to evaluate the viability using Live/dead staining. The change in viability as observed through BacLight staining, which enumerates live and dead cells, is shown in Figure 5b and Figure 6. Notably, clove oil does not show any significant increase in the population of dead cells. It is clear from Figure 5b and Figure 6 that the exposure to MF-1 resulted in maximum increase in the population of dead cells (41 ± 4%), followed by CHX, wherein 37 ± 3% of the cells died due to the exposure to 5 μg/mL of CHX. The exposure to MD-1 led to the death of 25 ± 2% cells.

### 2.4. Change in Biofilm Formation Activity through Quantitative and Qualitative Assay

**Figure 7:**
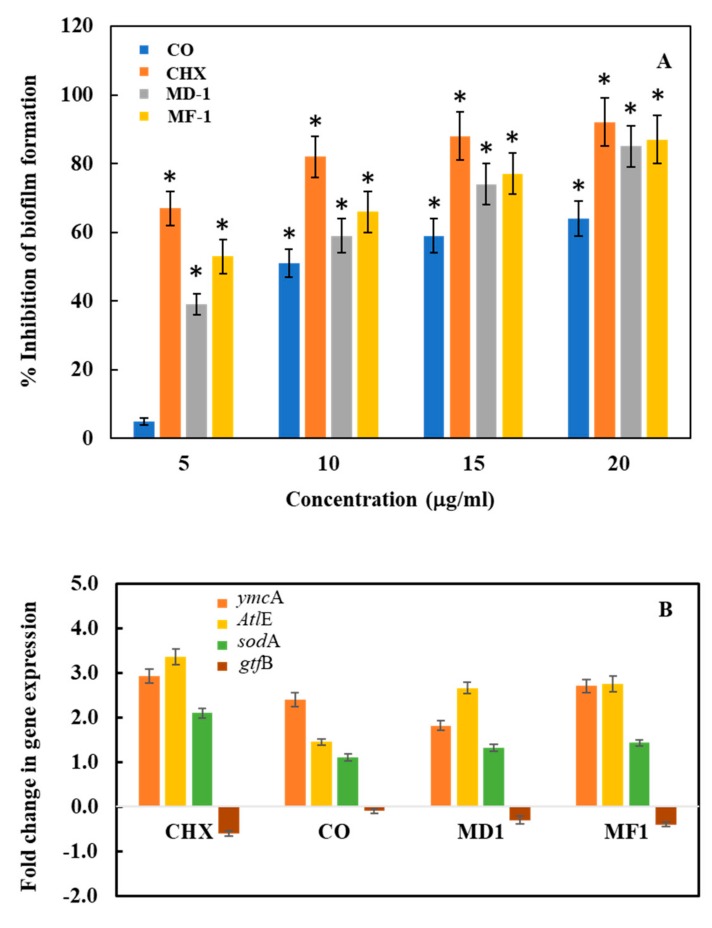
(**A**) Decrease in the *S. mutans* biofilm formation activity by various concentrations of the test compounds as determined by crystal violet assay. Change in the expression of *Atl*E, *gtf*B, *sod*A and *ymc*A genes of *S. mutans* when grown with the test compounds. Significant values (*p*-value > 0.05) are marked by asterisk. (**B**) The test compounds induced the expression of *Atl*E, *ymc*A and *sod*A genes, while *gtf*B gene involved in biofilm formation was downregulated.

**Figure 8:**
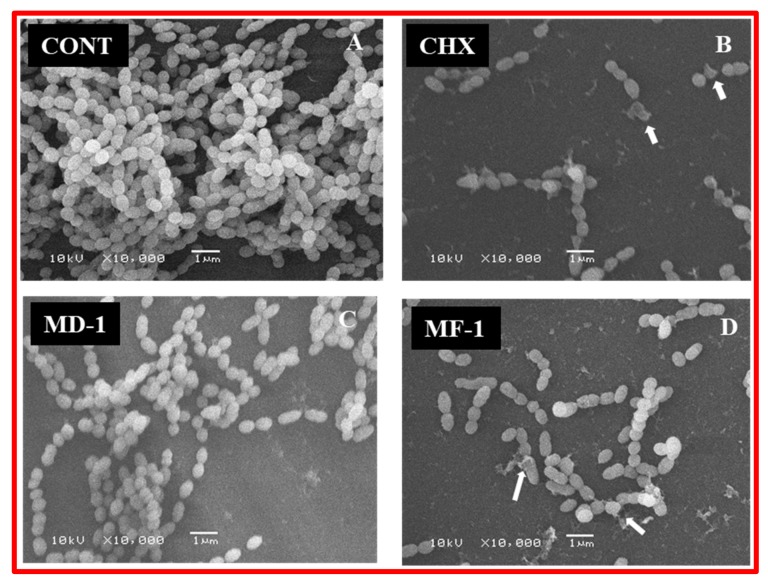
Reduction in the biofilm formation activity of *S. mutans* by chlorhexidine digluconate (CHX), and essential oils (MD-1 and MF-1) as visualized under SEM. The exposure to these test compounds results in lysed and deformed cells, as shown by arrows. While the cells in untreated control (CONT) appeared intact and dense.

The anti-biofilm formation activity of the test compounds was determined by using Crystal violet assay. Biofilm decreased by 97 ± 7%, 87 ± 6% and 82 ± 7% with 10 μg/mL of CHX, MF-1 and MD-1, respectively (Figure 7). Interestingly, clove oil also shows significant antibiofilm activity in CV assay with a decrease of 64 ± 5% at the highest test concentration. For qualitative assessment, *S. mutans* biofilms were grown in 48-well polystyrene plates with the test compounds and were subsequently visualized under a scanning electron microscope (Figure 8). Biofilm formation was not inhibited significantly by clove oil, as evident by the presence of well grown uniform biofilm with densely arranged bunches of cells, while, CHX, MF-1 and MD-1 inhibited the biofilm significantly, as evident from the presence of a few short chains of the cells (Figure 8). Furthermore, cells treated with MF-1, MD-1 and CHX appeared deformed and lysed and are indicated with the arrows in Figure 8.

### 2.5. Real-Time PCR Analysis

The influence of test compounds on cell death, biofilm formation activity and stress was evaluated by monitoring the change in the expression level of autolysin-like genes (*Atl*E- and *Atl*A-like genes), general stress gene (*Pnp*A), oxidative stress gene (*sod*A gene), and genes associated with biofilm formation (*ymc*A and *gtf*B genes). Since *Atl*A- and *Pnp*A-like genes were not amplified sufficiently by simple PCR using genomic DNA from *S. mutans* as template, these primers were not included for further RT-PCR studies. Moreover, the specificity of the primers was confirmed through the sequencing of amplified PCR products (Khan et al., 2017). The most significant change was recorded in the expression levels of Autolysin gene (*Atl*E), wherein a 3.36-, 2.66- and 2.76-fold increase was observed when *S. mutans* was grown in the presence of 10 μg/mL of CHX, MD-1 and MF-1, respectively (Figure 7B). This increased expression of *Atl*E gene indicates that the apoptosis-like activity in *S. mutans* is induced upon exposure to the test compounds. The expression of *ymc*A gene involved in the biofilm formation also increased by 2.93-, 1.81- and 2.71-fold in the presence of CHX, MD-1 and MF-1, respectively. On the contrary the expression of another gene involved in the biofilm formation *gtf*B decreased by −0.6-, −0.3- and −0.4-fold in the presence of CHX, MD-1 and MF-1, respectively, while, the expression of *sod*A genes increased by 1.1-, 1.32- and 1.43-fold when the bacteria were grown with clove oil, MD-1, and MF-1, respectively (Figure 7B).

## 3. Discussion

*S. persica* L. roots/stems known with various names like Mustard tree, Miswak, Koyojii and Pilu have been traditionally used for maintaining oral hygiene for centuries [3,4,6]. In this study, essential oils of dried (MD-1) and fresh (MF-1) roots of *S. persica* L. were isolated and the main components from the both oils were identified as benzyl isothiocyanate (MD-1; 68.1% and MF-1; 85.0%). The other major compounds in these essential oils were benzyl nitrile and benzaldehayde (Table 1). A high concentration of benzyl isothiocyanate (73.8%) in the essential oil from *S. persica* L. stem has also been reported previously [3,11], although the antimicrobial activity of compounds from *S. persica* L., especially against oral pathogens, including *Streptococcus mutans*, *Porphyromonas gingivalis*, *Tannerella forsythia*, and *Treponema denticola* [9,11], has been reported previously. The effects of drying on the chemical composition of essential oils from *S. perscia* L. roots and the resulting change in their antimicrobial activity have not been reported. In particular, detailed reports on the mechanisms leading to these antimicrobial activities and comparison with the antimicrobial activities of well-known commercial oral hygiene products Chlorhexidine digluconate and clove oil are not available. It is important to study the control of oral pathogens using plants and plant-based products as the excessive and unnecessary use of antibiotics has also led to the emergence of drug resistance in oral pathogens [16,17]. Furthermore, oral bacteria are emerging as a greater health threat than previously thought because of their involvement in other systemic diseases, in addition to oral diseases like periodontitis and gingivitis [18,19,20]. *S. mutans* is one such pathogen of great concern, which is a leading cause of dental caries and is also involved in other diseases [19,21]. The bacterium employs a number of mechanisms for its pathogenicity, including tolerance to pH, utilization of various carbon sources, biofilm formation and production of adhesins, proteases, haemolysins and others [21].

In this paper, EOs isolated from the dried and fresh roots of *S. persica* L. (MD-1 and MF-1) showed strong antibacterial and antibiofilm activities against *S. mutans*. MD-1 and MF-1 exhibit an IC_50_ value in the range of 10–11 µg/mL against *S. mutans*, which is comparable to those obtained for CHX (6 µg/mL). These values are much lower than the values reported earlier for the water and hexane extracts of *S. persica* L. [9] and comparable to those obtained for the essential oils against other oral pathogens including *Aggregatibacter actinomycetemcomitans* and *Porphyromonas gingivalis* [11]. In viability assays using live and dead staining and MTT assay, it was also found that the most effective essential oil was MF-1, which decreases the cellular viability by 80 ± 5% and increased the population of dead cells by 41 ± 4% at 20 and 10 µg/mL, respectively. These values were comparable to those obtained for CHX during this study. The mechanisms of the microbicidal activity of these EOs are not well understood. It has been demonstrated that benzyl isothiocynate, the main constituent of the tested essential oils, results in impaired respiration and intracellular protein aggregation in *Campylobacter jejuni*, resulting in the inhibition of various metabolic activities [22]. This is supported by the observations that in MTT assays metabolic activity of *S. mutans* cells decreased when exposed to essential oils (Figure 5A). It was also observed that exposure to essential oils results in the lysis of the cells and deformed cells (Figure 8). The RT-PCR studies of genes involved in the processes support these findings. The expression levels of autolysin like gene (*Alt*E) increased many folds upon exposure to essential oils (MD-1 and MF-1) and CHX, indicating that these test compounds induce apoptosis-like activity in *S. mutans*, resulting in decreased cell metabolism and viability. It has been reported in earlier studies that essential oils from *S. persica* L. cause cell wall damage in *S. enterica* [11]. Previously, we have also reported that essential oil compounds carvacrol and thymol cause apoptosis-like activity in *S. mutans* [14], which may be due to various reasons, including stress. The increased expression of *sod*A gene shows that exposure to the tested essential oils induces oxidative stress in *S. mutans*. Studies have demonstrated that isothiocynates can cause general stress in *E. coli*, as evidenced by the increased concentration of general stress regulator molecule guanosine penta/tetraphosphate ((p)ppGpp) [23]. Biofilm formation by oral pathogens is one of the most important traits contributing to their pathogenicity [19,24,25]. It was found that MD-1 and MF-1 both reduce the biofilm formation activities of *S. mutans* significantly, as biofilm formation was inhibited by 87 ± 6% and 82 ± 7%, in the presence of 10 µg/mL MD-1 and MF-1, respectively. A decrease in the expression of glycosyl transferase gene *gtf*B supports the observation that exposure to essential oils causes a decrease in biofilm formation. Benzyl isothiocynate is also known to inhibit biofilm formation in *Pseudomonas aeruginosa* [26]. This inhibition may be due to the inhibition of quorum sensing, which plays an important role in biofilm formation.

When the activities of MD-1 and MF-1 were compared, the latter exhibited higher antimicrobial activity. This is probably due to the comparatively higher concentration of benzyl isothiocyanate (Table 1) in MF-1. Among the three essential oils tested in this study, the least effective was clove oil. These results conclusively prove that the tested essential oil possess strong antimicrobial and antibiofilm activity against *S. mutans*, which is comparable to CHX. Therefore, benzyl isothiocyanate-rich essential oils from *S. persica* L. can serve as a good antimicrobial agent for oral hygiene and can also replace CHX, which, in addition to being an effective chemical antimicrobial agent, has many disadvantages such as its bitter taste, taste alteration for at least 4 h and tooth discoloration [15]. Other factors such as the toxicity of essential oils of *S. persica* L. rich in benzyl isothiocyanate and benzyl isothiocyanate alone should also be checked at realistic doses in human subjects before its commercialization. However, since it has been used traditionally for centuries with no known toxicity, the use of these essential oils for oral hygiene is highly recommended.

## 4. Materials and Methods

### 4.1. Plant Material

*S. persica* L. roots were procured in April 2013 from Batha (a local market) in Riyadh, Saudi Arabia (S.A.). The identification of plant species was carried out by a plant taxonomist at King Saud University. The specimen of plant species was preserved in a lab.

### 4.2. Isolation of EOs

Freshly collected *S. persica* L. roots were sliced to reduce their size and grinded in a coffee grinder to achieve the powdery form of the pant martial. A portion (3744.2 g) of grinded *S. persica* L. roots were subjected to shade air drying for three weeks to give 1872.6 g dried *S. persica* L. root. The grinded fresh (1453.4 g) and dried (653.2 g) roots of *S. persica* L. were separately hydro-distilled for 3 h in a conventional Clevenger apparatus [27]. The obtained yellow color oils were dried over Na_2_SO_4_ and kept at 4 ℃ in a refrigerator until they were analyzed. The oil yields from the fresh and dried *S. persica* L. roots were 0.2% and 0.03% (*w*/*w*), respectively.

### 4.3. Characterization of EOs Constituents

#### 4.3.1. GC and GC-MS Analysis of EOs

The essential oils from the fresh and dried *S. persica* L. roots were dissolved in analytical-grade diethyl ether (Sigma-Aldrich, Darmstadt, Germany) and an analysis was done using a GC-MS/GC-FID instrument outfitted with a HP-5MS (nonpolar) column. Analyses were performed using the same procedure as described earlier [28] which is given in detail in the Supplementary Materials File (S1).

#### 4.3.2. Linear Retention Indices (LRIs)

LRIs of essential oil constituents of the fresh and dried *S. persica* L. roots were calculated using the same procedure as that described earlier [28] and given in detail in the Supplementary Materials File (S2). The LRI values of each component are given in Table 1.

#### 4.3.3. Identification of EOs Constituents

Identification of fresh and dried roots essential oils constituents of *S. persica* L. was achieved through the analysis on a HP-5MS column using the same procedure as that described earlier [28] and given in detail in the Supplementary Materials File (S3).

### 4.4. Culture Media and Bacterial Strains

*Streptococcus mutans* ATCC 25175 obtained from American Type Culture Collection was used in the study. The strain was grown on brain heart infusion broth (Mast Group, Bootle, UK) with 2% sucrose (BHIS) until, unless mentioned otherwise. For longer storage and preservation, the strain was stored in 20% glycerol at −80 ℃.

### 4.5. Antimicrobial Activity against S. mutans

Spread plate and microdilution methods were used for determining the antimicrobial activity of the EOs MD-1 and MF-1. For determining the antimicrobial activity using a 96-well plate (microdilution method), 90 µL of BHIS broth was added to each well. Essential oils were dissolved and diluted in 5% DMSO. MD-1, MF-1, clove oil and chlorhexidine digluconate (CHX) were added to final concentrations of 0, 2, 4, 8, 10, 12, 14 and 16 µg/mL. CHX, a widely used antimicrobial agent in mouth wash and other oral hygiene products, was used as a positive control. Aliquots of 10 µL from overnight grown *S. mutans* culture were added to each well. Finally, the 96 well plate was incubated at 37 ℃ for 24 h. For determining the inhibition of colony forming units in the presence of essential oils (MD-1, MF-1 and clove oil) and CHX, aliquots of 500 µL from actively growing culture of *S. mutans* were added to tubes containing 5 mL BHIS broth. Essential oils and CHX were added to final concentrations of 0, 5, 10, 15 and 20 µg/mL to these tubes. The cultures were incubated on a shaker maintained at a speed of 150 rpm and a temperature of 37 ℃ for 12 h. After incubation, the CFU counts were determined by spreading 0.1 mL from appropriate dilutions of untreated and treated cells on BHI agar plates. The plates were finally incubated for 3 d at 37 ℃ and CFUs were counted and recorded.

### 4.6. Bacterial Viability

Two different assays were used for checking the bacterial viability, i.e., MTT assay and live and dead staining. MTT assay was performed to determine the change in bacterial viability upon treatment with the test compound [29]. BHIS broth containing 5, 10, 15 and 20 µg/mL of MD-1, MF-1, clove oil and CHX was used to grow *S. mutans* for 4–6 h at 37 ℃. Aliquots of 40 µL from each culture was then added in triplicates to the wells of the 96-well plates. To these wells, 10 µL of the MTT (Sigma, Ronkonkoma, NY, USA) solution prepared in PBS (5 mg/mL, pH 7.0) was added and the plates were incubated for 4 h at 37 ℃. An aliquot of 50 µL of lysis buffer containing 20% SDS in 50% DMF (pH 4.7) was added to each well and the plates were incubated overnight at 37 ℃. After incubation, pipetting was done to thoroughly mix the cells in each well and the absorbance at 570 nm was determined using a microtitre plate reader (Multiskan Ascent, Labsystems, Finland).

Live and dead staining was carried out by growing *S. mutans* with 5 µg/mL of MD-1, MF-1, clove oil and CHX at 37 °C for 8 h on a shaker. Following incubation, cells were centrifuged at 900× *g* for 10 min and were washed with PBS buffer. Cells were suspended again in sterile PBS (pH 7.5) and the BacLight™ Bacterial Viability Kit was used for performing Live and dead staining (LIVE/DEAD^®^, Molecular Probes, Eugene, OR, USA) according to the manufacturer’s protocol. After staining cells were visualized under a Nikon Eclipse 80i fluorescence microscope (Nikon Co., Tokyo, Japan). Cells appearing green and red in colour were recorded as total and dead cells, respectively. Ten fields were counted for every treatment and the population of live cells was calculated by subtracting the population of dead cells from the population of total cells in the same field. 

### 4.7. Antibiofilm Activity

#### 4.7.1. Crystal Violet Assay

The inhibition of biofilm formation activity of *S. mutans* by the test essential oils was evaluated using the standard protocol [30] on a 48-well polystyrene plate (Nunc, Roskilde, Denmark). To each well, 100 µL of *S. mutans* cells and 900 µL of sterile BHIS were added. Essential oils (MD-1, MF-1, and clove oil) and CHX were added to final concentrations of 5, 10, 15 and 20 µg/mL and plates were then incubated at 37 °C for 24 h. Floating cells and the medium were removed completely and gently using a micropipette. The biofilm attached to the bottom of the wells was washed gently three times with PBS (pH 7.4). To stain the biofilms, 500 µL of 0.4% crystal violet dye was added to each well and plates were incubated at room temperature for 15 min. Unbound dye was removed by washing the wells thrice gently with PBS. To solublize the crystal violet retained by the biofilm, 500 µL of 33% acetic acid was added to each well. A microtitre plate reader was used to record the absorbance at 620 nm.

#### 4.7.2. Scanning Electron Microscopy of Biofilms

Biofilms formed in the wells of polystyrene plates when grown with and without test compounds were visualized under a scanning electron microscope. For the assay, *S. mutans* was grown in the presence of 10 μg/mL of clove oil, MD-1, MF-1 and CHX. A micropipette was used to gently remove culture medium and to wash the biofilm with sterile PBS. Biofilms developed on the surface of polystyrene plates were stained at room temperature with 1% OsO_4_ fumes for 3 h and were finally washed thrice with milli-Q. A series of ethanol was used to dehydrate samples and subsequently SPI-Module line of modular sputter coater was used for the sputter coating of the sample with platinum. A magnification of 10,000× and an accelerating voltage of 10 KV was used to visualize the samples under a scanning electron microscope (JSM-6380; JEOL). For each sample, ten fields were observed and recorded.

### 4.8. Real-Time PCR Assay

The change in the expression levels of the six genes involved in biofilm formation, cell death and oxidative stress was determined using quantitative real-time PCR upon exposure to the test compounds. The genes included in the study are autolysin like gene *Atl*A and *Atl*E, involved in cell autolysis and cell-wall remodeling [21,31]. The other genes targeted in the study were involved in biofilm formation (*ymc*A and *gtf*B genes), and stress (Polyribonucleotide nucleotidyl transferase; *Pnp*A, superoxide dismutase; *sod*A), [32,33]. The primer sequences used in the study are given in the Supplementary Materials (Table S1). The effectiveness and specificity of the primers were checked by PCR amplification and subsequent sequencing of the PCR products. Cells were grown for 12 h at 37 °C in the presence of 10 μg/mL of essential oils (MD-1, MF-1 and clove oil) and CHX (6 μg/mL). The extraction of total RNA from untreated and treated cells, first strand cDNA synthesis, and real-time PCR were carried out as detailed in Khan et al. (2017).

### 4.9. Statistical Analysis

Two independent experiments were carried out each in triplicate and the results are presented as the mean ± SD. Unpaired t-test of GRAPHPAD PRISM version 5.0 (GraphPad Software, La Jolla, CA, USA) was used for statistical analysis. The level of statistical significance chosen was *p* < 0.05.

## 5. Conclusions

In this paper, the chemical constituents of EOs of fresh and dried *S. persica* L. roots were determined. The results show that the oil yields and contents of chemical constituents significantly differed in fresh and dried *S. persica* L. roots essential oils. Oil quantity and content of major bioactive compound benzyl isothiocyanate was found to be significantly higher in fresh *S. persica* L. roots oil. A comparative study on the antimicrobial and anti-biofilm activities of essential oils of fresh and dried roots of *S. persica* L. along with widely used oral hygiene agents CHX and clove oils revealed that essential oils from fresh root of *S. persica* L. significantly inhibit the growth, metabolism and biofilm formation activity of *S. mutans*. The activities were comparable to that of CHX, a widely used antimicrobial agent for oral hygiene. Since conventionally used chemotherapeutic agents are associated with multidrug resistance and other hazards such as toxicity, essential oils from *S. persica* L. can serve as a good alternative for maintaining oral hygiene as the source material is being traditionally used for the same purpose.

## Figures and Tables

**Table 1:** Chemical constituents identified from the fresh and dried roots essential oils of *S. persica* L.

No.	Compound	MF	MW	RT (min)	LRI_Lit_	LRI_Exp_^a^	MF-1 (%)	MD-1 (%)
1	Benzaldehyde	C_7_H_6_O	106	11.9	952	958	0.5	4.7
2	Benzyl nitrile	C_8_H_7_N	117	18.8	-	1140	13.4	26.0
3	Unknown	-	-	26.2	-	1355	0.2	0.6
4	Benzyl Isothiocyanate	C_8_H_7_NS	149	26.8	-	1373	85.8	68.1
	**Total identified**						**99.7**	**98.8**
	**Oil Yield**						**0.2**	**0.03**

MF, MW and RT denote molecular formula, molecular weight and retention time, respectively; LRI_Lit_ = Linear retention index from the literature (Adams, 2007); LRI^a^ = Computed LRI with reference to *n*-alkanes mixture (C8–C31) on HP-5MS; MF-1 = essential oil of *S. persica* L. fresh roots; MD-1 = essential oil of *S. persica* L. dried roots.

## Supplementary Materials

The following are available online at https://www.mdpi.com/2076-0817/9/1/66/s1, S1: Gas Chromatography (GC) and Gas Chromatography-Mass Spectrometry (GC-MS) Analysis of Essential Oils, S2: Linear retention indices (LRIs), S3: Identification of volatile components.
