# Supplementary material for: Antibiotic and Antibiofilm Activities of Salvadora persica L. Essential Oils against Streptococcus mutans: A Detailed Comparative Study with Chlorhexidine Digluconate
Source: Pathogens. 2020 Jan 16;9(1):66. doi: 10.3390/pathogens9010066 (PMC7169458; doi:10.3390/pathogens9010066)
Supplement: Supplementary file 1 [file pathogens-09-00066-s001.pdf]

Antibiotic and Antibiofilm Activities of *Salvadora persica* L. Essential oils: A Detailed Comparative Study with Chlorhexidine Digluconate

Merajuddin Khan[a], Hamad Z. Alkhathlan[a], Shams T. Khan*[b]

[a]*Department of Chemistry, College of Science, King Saud University, P.O.Box 2455 , Riyadh - 11451, Saudi Arabia.*

[b]*Department of Agricultural Microbiology, Aligarh Muslim University, Aligarh, 2002002, UP. India.*

*Corresponding Author. *E-mail:* shamsalig75@gmail.com (S. T. Khan).

S1. *Gas Chromatography (GC) and Gas Chromatography−Mass Spectrometry (GC-MS) Analysis of Essential Oils*

GC–MS was performed on an Agilent single-quadrupole mass spectrometer with an inert mass selective detector (MSD-5975C detector, Agilent Technologies, USA) coupled directly to an Agilent 7890A gas chromatograph which was equipped with a split–splitless injector, a quickswap assembly, an Agilent model 7693 autosampler and a HP-5MS fused silica capillary column (5% phenyl 95% dimethylpolysiloxane, 30 m × 0.25 mm i.d., film thickness 0.25 µm, Agilent Technologies, USA). The column was operated using an injector temperature of 250°C and the following oven temperature profile: an isothermal hold at 50°C for 4 min, followed by a ramp of 4°C/min to 220°C, an isothermal hold for 2 min, a second ramp to 280°C at 20°C/min and finally an isothermal hold for 15 min.

Approximately 0.2 µl of each sample diluted in diethyl ether (5% solution in acetone) was injected using the split injection mode; the split flow ratio was 10:1. The helium carrier gas was flowed at 1 ml/min. The GC–TIC profiles and mass spectra were obtained using the ChemStation data analysis software, version E-02.00.493 (Agilent). All mass spectra were acquired in the EI mode (scan range of m/z 45–600 and ionization energy of 70 eV). The temperatures of the electronic-impact ion source and the MS quadrupole were 230°C and 150°C, respectively. The MSD transfer line was maintained at 280°C for the nalysis. The GC analysis was performed on an Agilent GC-7890A dual-channel gas chromatograph (Agilent Technologies, USA) equipped with FID using nonpolar (HP-5MS) columns under the same conditions as described above. The detector temperature was maintained at 300°C for the analyses. The relative composition of the oil components was calculated on the basis of the GC–FID peak areas measured using the HP-5 MS column without using correction factor. Results are reported in Table 1 according to their elution order on the HP-5MS column.

S2. *Linear retention indices (LRIs)*

A mixture of a continuous series of straight-chain hydrocarbons, C8-C31 (C8-C20, 04070, Sigma-Aldrich, USA and C20-C31, S23747, AccuStandard, USA) was injected into nonpolar (HP-5MS) columns under the same conditions previously described for the oil samples to obtain the linear retention indices (LRIs) (also referred to as linear temperature programmed retention indices (LTPRI)) of the oil constituents provided in Table 1. The LRIs were computed using van den Dool and Kratz's equation.

S3. *Identification of volatile components*

The identification of different components of fresh and dried roots essential oils constituents of *S. persica* L. was done by matching their mass spectra with the library entries of mass spectra databases (WILEY 9th edition, NIST-08 MS library version 2.0 f, and Adams and Flavor libraries) as well as by comparing their mass spectra and linear retention indices (LRI) with published data [1-3].

Table S1: Inhibition of growth of *S. mutans* as observed in microdilution assays by recording the change in OD.

**Conc. (ug/ml)**	**OD_{620} nm**				
	**CHX**	**clove oil**	**MD-1**	**MF-1**	**Control**
14	0.068 ± 0.0053	0.325 ± 0.0091	0.071 ± 0.0040	0.073 ± 0.0055	0.359 ± 0.019
12	0.071 ± 0.0041	0.329 ± 0.0172	0.07 ± 0.0022	0.072 ± 0.0046	0.387 ± 0.022
10	0.07 ± 0.0033	0.331 ± 0.0115	0.072 ± 0.0041	0.071 ± 0.00332	0.369 ± 0.017
8	0.069 ± 0.0063	0.377 ± 0.0211	0.258 ± 0.0137	0.069 ± 0.00431	0.336 ± 0.023
6	0.088 ± 0.0051	0.402 ± 0.0203	0.397 ± 0.0172	0.115 ± 0.0065	0.414 ± 0.041
2	0.388 ± 0.0253	0.414 ± 0.0185	0.431 ± 0.0221	0.392 ± 0.023	0.369 ± 0.023
0	0.412 ± 0.0183	0.388 ± 0.0215	0.4 ± 0.025	0.387 ± 0.031	0.333 ± 0.025

Table S2: Details of primers used for RT-PCR studies.

S. No.	primer name	Seq (5'-3')	Gene	Product size (~bp)
1	AtlE_F<br>AtlE_R	agctggtcccaaaggaaatc<br>gcctgtgcccaataatcatc	*Atl*E	200
2	AtlA_F<br>AtlA_R	ggtttggaggcatcaactgt<br>agttggttggatatacgcgg	*Atl*A	138
3	Stress_F<br>Stress_R	taagccttacggtgcctttg<br>attaccaacatcgccatcgt	*Pnp*A	200
4	GtfB_F<br>GtfB_R	gccagccaatgttcatcttt<br>gaggcatttccccaaatgta	*gtf*B	160
5	YmcA_F<br>YmcA_R	gcttttgcaggaacatgaca<br>tggctcgataatcttggaca	*ymc*A	188
6	SodA39I_F<br>SodA39J_R	trcaycatgayaarcaccat<br>arrtartamgcrtgytcccaracrtc	*sod*A	400
7	16S_895F<br>16S_1100R	crcctggggagtrcrg<br>agggttgcgctcgttg	16S rRNA	200

1. Adams, R. Identification of essential oil components by gas chromatography/mass spectrometry. *Identification of essential oil components by gas chromatography/mass spectrometry.* **2007**.
2. Acree, T.; Arn, H. Flavornet. Cornell University, NYSAES. New York. 2012.
3. Linstrom, P.; Mallard, W. NIST Chemistry WebBook, NIST Standard Reference Database Number 69, June 2005. *National Institute of Standards and Technology, Gaithersburg MD* **2005**, *20899*.
